# Effects of team sports on sleep quality: a systematic review

**DOI:** 10.3389/fspor.2026.1746309

**Published:** 2026-06-02

**Authors:** Mohammad Mehdi Khaleghi, Fatemeh Ahmadi, Alireza Rabieezadeh, Josyula Tejaswi, Karuppasamy Govindasamy, Viorel Petru Ardelean, Vasile Emil Ursu, Vlad Adrian Geantă

**Affiliations:** 1 Department of Exercise Physiology, Faculty of Sport Sciences, Shahid Chamran University of Ahvaz, Ahvaz, Iran; 2 Persian Gulf Sports, Nutrition and Wellness Research and Technology Group, Persian Gulf University, Bushehr, Iran; 3 Department of Sport Science, School of Literature and Humanities, Persian Gulf University, Bushehr, Iran; 4 Symbiosis School of Sports Sciences, Symbiosis International (Deemed University), Pune, Maharashtra, India; 5 Department of Sports, Recreation and Wellness, Symbiosis International (Deemed University), Rangareddy, Telangana, India; 6 Department of Physical Education and Sport, Faculty of Physical Education and Sport, “Aurel Vlaicu” University of Arad, Arad, Romania; 7 Department of Physical Education and Sport, Faculty of Law and Social Sciences, University “1 Decembrie 1918” of Alba Iulia, Alba Iulia, Romania

**Keywords:** basketball, exercise, football, physical activity, pittsburgh sleep quality index (PSQI), soccer

## Abstract

**Introduction:**

Sleep disturbances are a prevalent global health concern with wide-ranging negative consequences. Although physical activity is recognized as a cost-effective strategy to enhance sleep quality, the specific impact of team sports such as football, basketball, handball, and volleyball—remains underexplored. This study systematically examines the effects of team sports on sleep quality.

**Methods:**

A comprehensive search strategy was used to retrieve all studies indexed in PubMed, Scopus, Embase, and Web of Science databases (up to June 11, 2024) with the Preferred Reporting Items for Systematic Reviews and Meta-Analyses (PRISMA) reporting guidelines. The search strategy incorporated keywords such as “Team sport*” OR “Teamsport*” OR “Handball” OR “Volleyball*” OR “Basketball*” OR “Netball*” OR “Basket ball” OR “football” OR “soccer” AND “Sleep Qualit*”.

**Results:**

Out of 1,148 initially identified records, 491 studies were screened, and 11 met the inclusion criteria for review. These studies involved 809 participants (367 males, 442 females) aged 13.5–65 years, including adolescents, college students, elite athletes, and untrained individuals. Findings showed that soccer, Zumba, volleyball, and handball interventions significantly improved sleep quality, while results for basketball were inconsistent college players benefited, but elite and wheelchair athletes showed no significant changes.

**Conclusion:**

Participation in team sports such as basketball, football, Zumba, handball, and volleyball appears to be associated with improvements in sleep quality. However, given the limited number of included studies and the heterogeneity in study designs, populations, and outcome measures, these findings should be interpreted with caution. Overall, the current evidence suggests a potentially positive relationship between engagement in team sports and sleep outcomes, but further well-designed and large-scale studies are needed to establish the strength and generalizability of this association.

**Systematic Review Registration:**

https://www.crd.york.ac.uk/PROSPERO/view/CRD42024557907, identifier CRD42024557907.

## 1 Introduction

Sleep, as a fundamental component of a healthy lifestyle, engages in complex interactions with other lifestyle factors. Neglecting any one of these components may adversely influence the others and, consequently, compromise overall health (1). Poor sleep quality and insufficient sleep are widely recognized as global public health concerns associated with a range of negative health outcomes (2). Epidemiological evidence suggests that at least one in five adults experiences chronic sleep disturbances (3, 4). Moreover, sleep-related problems are not confined to the general population; athletes are also affected. Research indicates that, compared with non-athletes, athletes often experience shorter sleep duration, more frequent nocturnal awakenings, prolonged sleep onset latency, and reduced sleep efficiency (1, 5–8). Insomnia symptoms and heightened sleep sensitivity have likewise been reported as prevalent among professional athletes (5, 7, 8).

In the general population, sleep disorders and poor sleep quality have been shown to contribute to the accumulation of fatigue, excessive daytime sleepiness, and mood disturbances (9), as well as to the onset and progression of various health conditions, including overweight and obesity (10), infectious diseases (11, 12), cardiovascular disorders, depression, and cancer (13–15). Similarly, in athletic populations, repeated exposure to high-intensity training and competition schedules—often accompanied by substantial physiological and psychological stress—elevates recovery demands. Consequently, the overall need for sleep, as a critical component of effective recovery, may be increased in athletes (1). Evidence further suggests that sleep disturbances can negatively affect training adaptation, recovery processes (16), and both physical and functional injury risk, ultimately impairing athletic performance (9, 17). Therefore, promoting sufficient and high-quality sleep among athletes appears essential for protecting them against a range of physiological impairments and for optimizing sport performance outcomes (17).

Sleep quality is defined by an individual's overall satisfaction with key dimensions of sleep, including efficiency, timing, duration, and awakenings after sleep onset (2). It is influenced by a range of factors, such as age, gender, marital status, level of physical activity, retirement status, comorbidity burden (18), dietary patterns (19), genetic predisposition (20), and environmental conditions (21). Accordingly, good sleep quality may be regarded as a robust predictor of physical and mental health, vitality, and overall well-being (22).

Since team sports athletes have been found to have maladaptive pre-sleep behaviors and poorer sleep characteristics than individual sports athletes (23); and the specific effects of soccer (football), basketball, handball, and volleyball team sports on sleep quality have not been summarized. Therefore, the authors decided to conduct a study with the aim of a comprehensive and systematic investigation of the impact of team sports on the quality of sleep of athletes involved in these sports.

## 2 Methods

### 2.1 Protocol and registration

This systematic review complied with the Preferred Reporting Items for Systematic Reviews and Meta-Analyses (PRISMA) reporting guidelines (24, 25) and ethical standards (26) of sports and exercise science research. This study was registered under number CRD42024557907 in the international prospective systematic review database.

### 2.2 Data sources and search strategies

One thousand and one hundred forty-eight articles from PubMed (MEDLINE), Scopus, Embase, and Web of Science were obtained on June 11, 2024 using the keywords “Team sport*” OR “Teamsport*” OR “Handball” OR “Volleyball*” OR “Basketball*” OR “Netball*” OR “Basket ball” OR “football” OR “soccer” AND “Sleep Qualit*” with no limitations on language or publication year.

### 2.3 Eligibility criteria

Eligibility was determined using the PICOS structure (Population, Intervention, Comparison, Outcomes, Study design) (27), delineated as follows: (1) population: All participants in various team sports such as football, volleyball, basketball, handball and other team sports; (2) intervention: Perform various team sports such as football, volleyball, basketball, handball and other team sports; (3) comparison: With or without control group; (4) outcomes: Sleep quality; and (5) study Design: All types of studies with different study designs.

The exclusion criteria included: content unrelated to the topic, reviews and case reports, conference papers, editorials, erratum and retracted papers, lack of access to full-text articles, and the presence of any additional interventions beyond the implementation of team-based exercises.

### 2.4 Study selection

Both reviewers, MM KH and F A, independently conducted the initial and subsequent screenings using a two-stage process. The identical records were removed manually. A third reviewer (V.E.U.) settled the disagreements among the reviewers. The relevance of sleep quality to team sport was the standard for second-stage screening. Data extraction and quality assessment.

Relevant data were extracted from eligible studies following the screening process using a predefined extraction template. This template includes author names, publication years, the location of the study, the main title of the article, the name of the journal, the study design, age and gender of participants, sample size, measurement tools, interventions, and research outcomes. The articles’ methodological quality was assessed using the Mixed Method Appraisal Tool (MMAT-version 2011) (28, 29).

## 3 Results

### 3.1 Search selection and inclusion of publications

**Figure 1:**
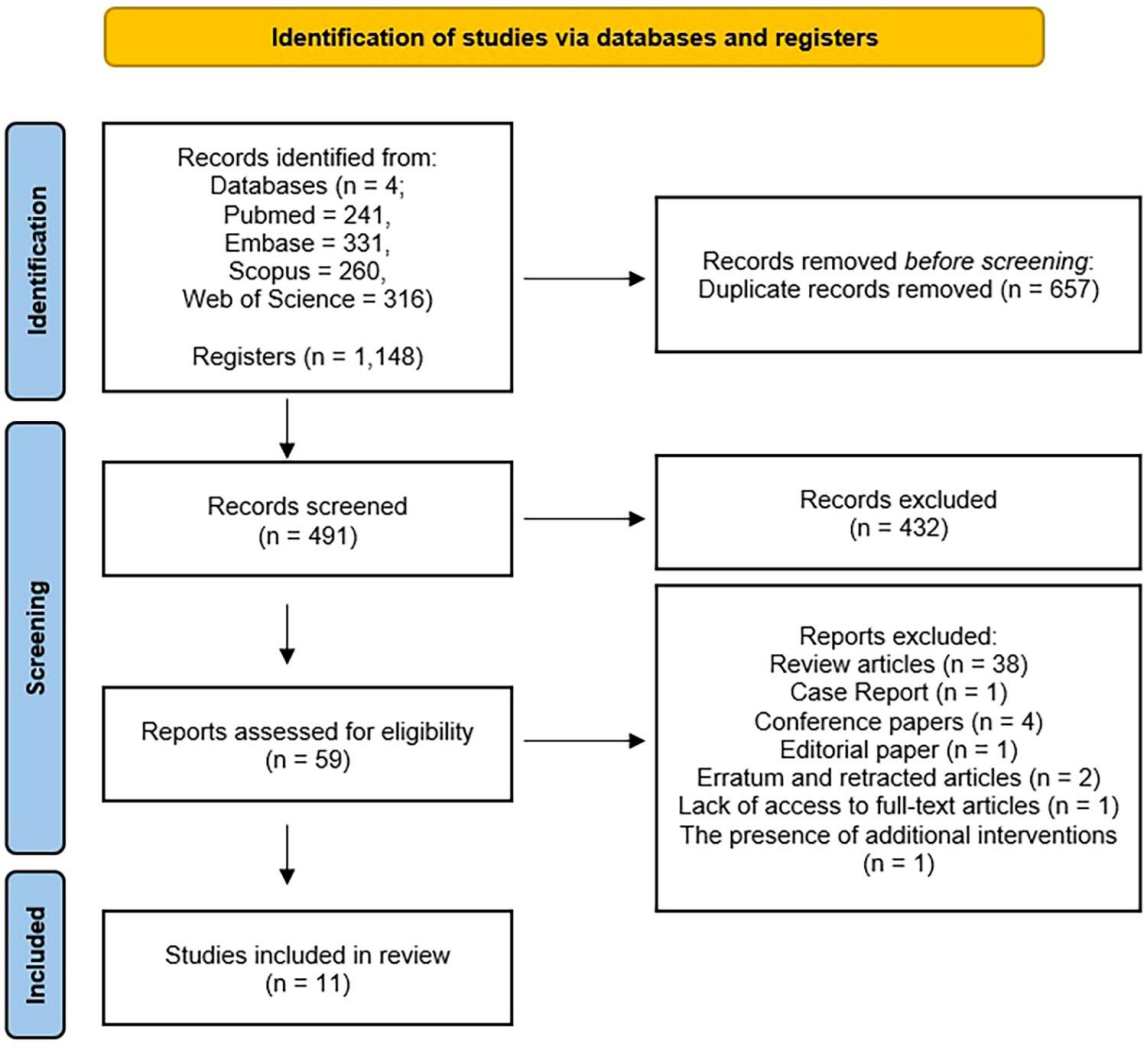
PRISMA flow diagram.

A total of 1,148 articles were identified through searches of four electronic databases. Of these, 657 articles were automatically removed as duplicates upon transfer to the bibliography management software (EndNote). From the remaining 491 articles for title and abstract screening, 432 non-relevant articles, 38 review articles, one case report, four conference papers, one editorial, and two erratum and retracted articles were excluded. In addition, one article was excluded due to lack of access to its full text, and another was removed because it included additional interventions beyond the intended scope of team-based exercises. Ultimately, 11 articles were comprehensively analyzed and included in the study (Figure 1). The PRISMA diagram outlines the process of selecting articles from the databases, as illustrated in the accompanying flowchart.

### 3.2 Characterization of studies

In total, the studies included a cumulative sample of 809 participants, consisting of 367 males and 442 females. The age range of participants varied broadly across studies, spanning from adolescents to professional and elite adult athletes (age range = 13.5–65 years), with most participants falling within the adolescent to young adult age groups. The included populations were diverse and encompassed various demographic groups such as elite and professional players (30–33), male adolescent (34, 35), male junior (36), female hospital employees (37), untrained male students (38), and college students of both genders (39, 40) (Table 1).

**Table 1:** Characteristics of the included studies.

Authors/year	Country	Study design	Participants	Sleep quality measurement tool	Type and duration of team sports	Main results
López-Flores et al. (33)	Spain	Interventional study	10 male Spanish elite wheelchair basketball athletes (Mean age = 35 ± 8 years)	The ActiGraph wGT3X-BT triaxial accelerometer, along with self-reported sleep diaries documenting bedtime and wake time	Wheelchair basketball 5 weeks	No significant differences on sleep quantity and quality
Frytz et al. (34)	Austria	Observational study	53 male adolescent soccer players (under 15 year (*n* = 45) and under 16-year elite teams (*n* = 8) (Mean age = 14.36 ± 0.55 years)	Pittsburgh sleep quality index (PSQI), Electrocardiography (ECG) and actigraphy devices	Soccer training and games in academy at least three consecutive days following their habitual training schedules 14 days	Higher training intensity was associated with increased wake time, while later training sessions resulted in longer sleep duration. Additionally, engaging in one training session per day was most beneficial for sleep quality.
Saidi et al. (30)	Tunisia	Interventional study	14 elite soccer players were evaluated 3 times (T1, T2, and T3) over 12 weeks (T1–T2: 6-wk regular period of match play and T2–T3: 6-wk congested period of match play) (Mean age = 20.9 ± 0.8 years)	Wellness Status Evaluation (Hopper Index)	Soccer match play five times per week with one match for 12 weeks within the competitive season	No significant alteration in the sleep score
Barene and Krustrup (37)	Norway	Interventional study	107 female hospital employees [Football group (FG): *n* = 37, Zumba group (ZG): *n* = 35, and Control group (CG): *n* = 35] (Aged range: 25–65 years)	Self-reported sleep problems during the past three months were assessed using the following four single-items derived from a modified version of the Karolinska Sleep Questionnaire	Football and Zumba During the first 12 weeks, both intervention groups were offered three 1 h training sessions per week, with the opportunity of two 1 h sessions during the last 28 weeks. For 40 weeks	FG and ZG improved sleep outcomes
Ji et al. (39)	China	Comparative study	197 college students (115 males and 82 females) In three groups: team sports, individual sports, and CG (Mean age = 21.3 years)	PSQI	Basketball games twice a week for 6 weeks	Team sports improved sleep quality compared to the CG.
Tomar and Allen (38)	Saudi Arabia	Interventional study	24 untrained male students intervention group (*n* = 14, 19.78 ± 1.05 years) and CG (*n* = 10, 19.60 ± 0.96 years)	PSQI	Small sided recreational handball for 12 weeks	Improved in sleep quality
Rodrigues et al. (31)	Brazil	Interventional study	19 youth soccer players (mean age of 17 ± 1 years)	PSQI	Soccer training for 4 weeks	Improved in sleep duration and quality
Johnston et al. (40)	China	Quasi-experimental study	291 college students [team sports classes: *n* = 138, (71 males and 67 females; mean age: 18.94 ± 1.28 years) and aerobic dance classes: *n* = 153 females; mean age: 18.34 ± 1.20 years]	PSQI	Volleyball or soccer A 90 min session per week for 12 weeks	Significant improved in sleep quality
Chou et al. (35)	Taiwan	Observational study	9 elite male adolescent Division I high school basketball players (Aged range: 15–18 years)	PSQI	Basketball during the national competitive season (National High-School Basketball Competition Series, High School Basketball League, Taiwan) and again at the end of the off-season recovery period	The sleep quality did not differ between periods.
Selmi et al. (32)	Tunisia	Interventional study	15 professional soccer players (Mean age = 24 ± 1 years)	Well-being indices questionnaire	Soccer For 6 weeks	Significant increase in sleep quality
Brand et al. (36)	Switzerland	Observational study	70 Male junior chronic and intense football players (CIFP; *n* = 36; mean age: 15.42 ± 0.86 years) and Age-matched male controls (CG; *n* = 34; mean age: 15.30 ± 0.85 years)	PSQI, the German adaptation was taken from a conventional and widely used manual for psychological treatment of sleep complaints	Football	Improved in sleep onset latency, awakenings, sleep quality, and sleep patterns

PSQI, pittsburgh sleep quality index; ECG, electrocardiography; FG, football group; ZG, Zumba group; CG, control group.

The included studies were conducted across multiple continents, involving countries from Europe [Spain (33), Austria (34), Norway (37), Switzerland (36)], Africa [Tunisia (30, 32)], Asia [China (39, 40), Saudi Arabia (38), Taiwan (35)], and South America [Brazil (31)]. These studies examined a variety of team sports, including wheelchair basketball (33), basketball (35, 39), soccer (30–32, 34, 36), football and Zumba (37), small-sided recreational handball (38), and volleyball or soccer (40). The types of included studies were as follows: five interventional studies (30–32, 37, 38), four observational studies (33–36), one comparative study (39), and one quasi-experimental study (40) (Table 1).

The 11 included articles were published in reputable academic journals, including Sleep Science (33), Life (34), International Journal of Sports Physiology and Performance (28), Behavioral Sciences (39), International Journal of Environmental Research and Public Health (35, 37), Medical Science (38), Motriz: Physical Education Magazine (31), Journal of American College Health (40), European Review of Applied Psychology (32), and Journal of Health Psychology (36).

The methodological quality of the included studies was assessed using the MMAT, and although most studies met the five core criteria with “Yes” ratings—indicating acceptable baseline methodological standards—this should not be interpreted as uniformly high quality. Despite heterogeneity in study design, populations, and intervention characteristics, two studies exhibited a potentially elevated risk of nonresponse bias, which may affect the representativeness of their findings. Additionally, several studies relied on self-reported measures, lacked robust control conditions, or included small sample sizes, all of which may introduce bias and limit the strength of the evidence. Variability in intervention protocols and outcome assessments further constrains comparability across studies. Therefore, while the included studies were eligible based on MMAT criteria, their methodological limitations warrant cautious interpretation of the overall evidence (Table 2).

**Table 2:** The quality assessment of the included studies using the mixed methods appraisal tool (MMAT).

Quantitative studies					
Study	1.1. Is the sampling strategy relevant to address the research question?	1.2. Is the sample representative of the target population?	1.3. Are the measurements appropriate?	1.4. Is the risk of nonresponse bias low?	1.5. Is the statistical analysis appropriate to answer the research question?
López-Flores et al. (33)	Yes	Yes	Yes	Yes	Yes
Frytz et al. (34)	Yes	Yes	Yes	No	Yes
Saidi et al. (30)	Yes	Yes	Yes	No	Yes
Barene and Krustrup (37)	Yes	Yes	Yes	Yes	Yes
Ji et al. (39)	Yes	Yes	Yes	Yes	Yes
Tomar and Allen (38)	Yes	Yes	Yes	Yes	Yes
Rodrigues et al. (31)	Yes	Yes	Yes	Yes	Yes
Johnston et al. (40)	Yes	Yes	Yes	Yes	Yes
Chou et al. (35)	Yes	Yes	Yes	Yes	Yes
Selmi et al. (32)	Yes	Yes	Yes	Yes	Yes
Brand et al. (36)	Yes	Yes	Yes	Yes	Yes

### 3.3 Outcome measurement

The included studies assessed a range of sleep-related parameters. Specifically, they evaluated sleep quantity and quality (33, 36), sleep quantity and sleep architecture (34), sleep quality (30–32, 35, 38–40), sleep problems (37), and sleep duration (31). These assessments provide a comprehensive overview of various dimensions of sleep in relation to team sports participation (Table 1).

### 3.4 Team sport protocols

Various protocols were implemented in the included studies to administer team-based interventions, as described below.

#### 3.4.1 Basketball

##### 3.4.1.1 Wheelchair basketball

The intervention spanned five consecutive weeks. During the first three weeks, participants engaged in regular training sessions and competitive games. In the fourth week, training sessions continued alongside two playoff games, while in the fifth week, only training sessions were held without any games. Each week included three training sessions scheduled from 6:30 to 8:30 p.m., with an average duration of 120 min per session (33).

##### 3.4.1.2 Basketball games

Participants in team sports group performed basketball sessions structured as full games. Each session began with a 10-min warm-up, followed by a 40-min basketball game conducted according to standard rules. Participants were divided into smaller teams to compete against one another. The final 10 min of each session were dedicated to cool-down and group discussion (39).

Another study examined basketball training during two distinct periods: the national competitive season (National High-School Basketball Competition Series and High School Basketball League in Taiwan) and the end of the off-season recovery period. During both periods, athletes followed a structured program comprising basketball-specific skill training and strength and conditioning exercises, supervised by their coaches. As part of their routine, Division I high school basketball players in Taiwan continued regular training through the summer break. For research purposes, physical performance was assessed at two points: within three days following the last playoff game (pre-test) and three months after the end of the competitive season (post-test). To minimize the influence of prior exertion on testing outcomes, participants refrained from strenuous exercise and resistance training for two days before the post-test assessment during the off-season recovery period (35).

#### 3.4.2 Soccer (football)

In one study, participants underwent a structured training regimen spanning 12 weeks during the competitive season. Players trained five times per week and participated in one official match per week. Each training session lasted approximately 90 ± 8 min and was conducted in the afternoons from 3:30 to 5:00 PM (30).

Another study implemented a four-week multicomponent training program that integrated technical and tactical drills, resistance training, aerobic treadmill workouts, interval training, and repeated sprint exercises. Monday (Power Training): Exercises included Leg Press 45°, Smith Squat, Leg Extension, Leg Curls, Hip Abduction/Adduction, Calf Raises—performed in 3 sets of 15 repetitions at 60% 1-RM with 45-s rest intervals. Athletes were instructed to execute movements at maximal speed. Following this, 20 × 20-m sprints were performed with 30-s rest intervals. Tuesday (Recovery Power Training): Volume and intensity were reduced to facilitate recovery for the next day's ball training. Athletes performed 3 sets of 8 repetitions at 40% 1-RM with 60-s rest intervals, followed by 80 min of treadmill running at 10–12 km/h. Wednesday to Friday (Ball Training and Interval Workouts): Each day included 90 min of technical drills, small-sided tactical games, and interval training (1:1 work-to-rest ratio). Athletes ran laps in a 100 × 50 m field, completing each lap in 1 min followed by 1 min of passive rest, repeated 10 times. Each session concluded with stretching exercises (31).

In another study, a six-week pre-season soccer training program was divided into a 2-week basic training (BT) phase and a 4-week intensified training (IT) phase. Basic Training (BT): Included 6–7 training sessions per week focused on aerobic conditioning and muscular strength development through weight training. No matches were played during this period. Intensified Training (IT): Included 9–10 training sessions per week, one friendly match, and one rest day. Sessions incorporated continuous running at 70%–80% HRmax, interval running at 90%–95% HRmax, small-sided games at high intensity, repeated sprints (15–30 m), speed and strength exercises (e.g., jumps, elastic resistance), technical and tactical drills, and simulated match play (32).

One study simply reported soccer participation within a football club setting, without detailed information regarding the structure or content of the training sessions (36). Another study also referred to soccer training and game participation within an academy environment but provided limited protocol specifics (34).

#### 3.4.3 Football and Zumba

In one study, during the initial 12 weeks of the intervention, both groups participated in three 1-h training sessions per week. In the subsequent 28 weeks, participants had the option to attend two 1-h sessions per week. The football training was conducted in the form of small-sided games, held either in a traditional gymnastics hall at the hospital (10 m × 20 m) or in a nearby municipal sports hall (20 m × 40 m). The Zumba sessions were conducted at a fitness center close to the hospital (37).

#### 3.4.4 Small sided recreational handball

In one of the included studies, the team-based physical activity intervention involved a 12-week supervised small-sided recreational handball program for participants in the intervention group. A total of four teams were formed, each consisting of four players, deviating from the standard seven-player format. All games were conducted on a standard handball court measuring 40 × 30 m. Sessions were held in the evening, twice per week, with each session lasting 30 min. Each session began with a 10-min warm-up that included jogging and handball-specific drills, and concluded with a 10-min cool-down consisting of stretching and relaxation exercises (38).

#### 3.4.5 Volleyball or soccer

In one of the included studies, participants in the team sports group engaged in either volleyball or soccer. Both groups took part in one 90-min physical education session per week over a 12-week period. In addition to the scheduled sessions, students were instructed to independently complete a total of 68 km of running throughout the 12 weeks (40).

### 3.5 Main results

#### 3.5.1 Basketball

Three studies included in the present review independently demonstrated various effects of basketball participation on sleep outcomes.

##### 3.5.1.1 Wheelchair basketball

López-Flores et al. found that wheelchair basketball did not significantly impact sleep quantity or quality. Their study reported no significant differences in sleep-related parameters across the pre-playoff, playoff, and post-playoff weeks (*p* > 0.05), and no significant associations were observed between training load, sleep metrics, and recovery values (33).

##### 3.5.1.2 Basketball games

Two studies included in the present review independently demonstrated the effects of team basketball on sleep-related outcomes. Ji et al. (39) reported that the rate of improvement in sleep quality was 28.4% in the control group, 78.1% in the individual sports group, and 78.8% in the team sports group. Although both individual and team sports significantly improved sleep quality among college students, no significant difference was observed between the two groups (OR = 7.32 vs. 7.98, *p* = 0.21), indicating comparable effectiveness.

However, Chou et al. found no significant differences in overall sleep quality scores or its components—such as sleep latency, duration, efficiency, disturbances, use of sleep medication, or daytime dysfunction—between the competitive season and off-season periods in elite male basketball players [competitive season (CS): 6.2 ± 0.9 vs. off-season (OS): 6.9 ± 1.1 AU; *p* > 0.05] (35).

#### 3.5.2 Soccer (football)

Five studies included in the present review independently demonstrated various effects of soccer participation on sleep outcomes.

Frytz, et al. investigated the impact of team-based football training on various sleep-related parameters. Their findings indicated that nights following the most intense training sessions were associated with significantly higher wake time from the first to last sleep epoch (WTSP) (*U* = 368.000, *Z* = −2.264, *p* = 0.024) and showed trends toward increased wake after sleep onset (WASO) (*U* = 397.000, *Z* = −1.892, *p* = 0.058) and a higher number of prolonged awakenings (*U* = 402.000, *Z* = −1.857, *p* = 0.063) compared to nights after moderate training sessions. However, no significant differences were observed in total sleep duration or sleep onset latency. Regarding the timing of training, later training sessions before bedtime led to significantly longer sleep periods (TSP) (*U* = 142.500, *Z* = −2.336, *p* = 0.020) and total sleep time (TST) (*U* = 151.500, *Z* = −2.125, *p* = 0.034) compared to earlier sessions. Despite this, the timing of training did not significantly correlate with perceived training intensity or overall sleep quantity.

When comparing different training frequencies, nights following one training session were associated with significantly greater total sleep time (*U* = 620.500, *Z* = −2.183, *p* = 0.029), increased REM sleep (*U* = 583.000, *Z* = −2.524, *p* = 0.012), and decreased NREM sleep (*U* = 583.000, *Z* = −2.524, *p* = 0.012) compared to rest days. A trend toward higher WTSP was also observed after two training sessions. Overall, actigraphy-derived sleep quantity did not significantly differ across training intensities or frequencies, although nap frequency and duration varied slightly depending on the training schedule (34).

Saidi et al. (30) reported no significant changes in sleep scores across different competitive soccer periods. Rodrigues et al. (31) observed significant improvements in both sleep duration (*p* = 0.03) and sleep quality (*p* = 0.02) following a soccer-based intervention. Selmi et al. (32) found that participation in soccer training significantly enhanced sleep quality (*p* < 0.001). Brand et al. reported that chronic and intense football players (CIFPs), compared to controls, exhibited better mood (both morning and evening), improved sleep quality, enhanced restorative sleep, shorter sleep latency, fewer awakenings after sleep onset, and longer total sleep time. Notably, these beneficial effects were consistent across both school and off-school nights. Furthermore, interaction effects revealed that CIFPs experienced a decrease in the number of awakenings and an increase in restorative sleep from school to off-school nights, whereas the control group showed opposite trends (36).

#### 3.5.3 Football and Zumba

One study included in the present review investigated the effects of team-based physical activities—football and Zumba—on sleep outcomes. The results showed that the Zumba group experienced a significant reduction in the frequency of poor and restless sleep over the 40-week intervention compared to the control group (*p* = 0.004), while no significant between-group difference was observed for the football group (*p* = 0.296). However, both the Zumba and football groups demonstrated significant within-group improvements in poor and restless sleep from baseline to 40 weeks (*p* < 0.001 and *p* < 0.05, respectively).

The Zumba group also showed trends toward improved sleep initiation and reduced total sleep problems, with significant within-group improvements from baseline to both 12 and 40 weeks (*p* < 0.001). In contrast, the football group only showed a significant within-group reduction in difficulties falling asleep (*p* < 0.05), without significant change in total sleep problems.

No significant between-group differences were observed regarding early awakenings or waking up several times during the night. Nevertheless, the Zumba group showed a tendency for reduced early awakenings at 12 weeks (*p* < 0.1) and significant within-group improvements in night awakenings with difficulty returning to sleep at both 12 (*p* < 0.01) and 40 weeks (*p* < 0.05) (37).

#### 3.5.4 Small sided recreational handball

In the study by Tomar and Allen, participation in small-sided recreational handball resulted in a significant improvement in sleep quality, as evidenced by a statistically significant difference between the intervention and control groups following the intervention (*t*₍_22_₎ = 3.776, *p* = 0.001), with the intervention group reporting a lower mean sleep score (9.28 ± 10.5) compared to the control group (10.5 ± 2.12) (38).

#### 3.5.5 Volleyball or soccer

In the study conducted by Johnston et al., participation in team sports such as volleyball or soccer was found to improve multiple dimensions of sleep quality. All seven components of the Pittsburgh Sleep Quality Index (PSQI)—including subjective sleep quality, sleep latency, sleep duration, habitual sleep efficiency, sleep disturbances, use of sleep medications, and daytime dysfunction—were assessed. Results showed that from pre-test to post-test, components one through three (subjective sleep quality, sleep latency, and sleep duration), as well as components six and seven (use of sleep medications and daytime dysfunction), improved in the team sports group. Components four and five (habitual sleep efficiency and sleep disturbances) also demonstrated decreased across both intervention groups.

The global PSQI score decreased significantly in the team sports group from a pre-test mean of 5.58 (SD = 2.69) to a post-test mean of 3.82 (SD = 2.62), indicating a shift from poor to good sleep quality. A two-way mixed-design ANOVA revealed a significant main effect of time on global PSQI scores (*F* = 121.023, *p* < 0.001, *η*^2^ = 0.298), confirming that sleep quality significantly improved regardless of group assignment. However, there was no statistically significant interaction between the type of physical activity and PSQI outcomes (*F* = 3.213, *p* = 0.074), suggesting that the observed improvements were not significantly different between the team sports and aerobic dance groups.

Further ANOVA analyses of individual PSQI components demonstrated that sleep latency and sleep duration significantly improved over time (*p* < 0.001 for both), while daytime dysfunction also showed both a significant time effect (*F* = 8.071, *p* = 0.005) and a significant group effect (*F* = 4.138, *p* = 0.043), favoring the intervention. These findings support the beneficial impact of team sports such as volleyball and soccer on various aspects of sleep health (40).

## 4 Discussion

The present systematic review synthesizes the available evidence on the association between participation in team sports and sleep quality. Overall, most studies indicate a positive relationship between engagement in team-based activities—such as football, basketball, Zumba, handball, and volleyball—and improvements in sleep outcomes, with only four of the eleven included studies reporting no significant effects. However, this general trend should be interpreted cautiously due to substantial clinical and methodological heterogeneity across the studies.

The included studies differed markedly in participant characteristics (e.g., age, gender, health status, baseline sleep quality), intervention features (e.g., type of sport, training intensity, frequency, and duration; recreational vs. competitive settings), and study design (e.g., cross-sectional vs. interventional). Additionally, sleep outcomes were assessed using both subjective and objective measures, contributing to variability in findings. Notably, more consistent improvements were observed in studies employing structured, regular team sport interventions. Collectively, these differences limit comparability and the strength of conclusions; thus, the evidence should be considered indicative rather than definitive, warranting a nuanced interpretation.

Regular exercise has been shown to improve sleep quality and promote overall health (41, 42). A new meta-analytic review has reported that engaging in combined exercise routines as well as resistance training is significantly associated with sleep quality in the older population with insomnia. However, the same study indicated that aerobic physical activity was associated with a notable decline in their sleep quality (43). Another meta-analysis showed that exercise interventions shorter than 12 weeks improved sleep quality and daytime dysfunction, while interventions lasting 12 weeks or more reduced the use of sleep medications in women with insomnia symptoms (44). A study reported that 12 weeks of high-intensity interval training significantly improved sleep quality in adults (45). Similarly, Lazaridou et al. found that yoga practice effectively enhanced sleep quality among participants (46).

Evidence suggests that regular physical activity, regardless of type, significantly improves sleep quality and reduces fatigue over time (47). Exercise helps regulate the sleep–wake cycle by temporarily increasing body temperature, which subsequently drops and induces sleepiness (48). Additionally, physical activity may alleviate mental tension and stabilize mood, facilitating a smoother cognitive and emotional transition into sleep (48).

Despite evidence supporting the positive effects of various forms of exercise on sleep, no prior studies, to the authors’ knowledge, had specifically examined the impact of team sports on sleep quality. The present review addressed this gap and found that participation in team sports such as football, basketball, Zumba, handball, and volleyball was generally associated with improved sleep quality.

In this context, physical exertion involved in team and ball sports, induces bodily fatigue and subsequently enhances sleep quality (39). However, some studies have reported that participation in team sports does not significantly alter sleep quality. This may be attributed to factors such as athletes’ prior experience with competition, performing the activity in familiar environments (33), or individual differences in physiological conditions and sleep patterns influenced by age and gender (49). In some cases, high-intensity training or competition, late scheduling of sessions, and multiple practices or matches within a short period may hinder adequate physical recovery, potentially leading to somatic discomfort. Combined with competitive anxiety, these factors may contribute to unchanged or even reduced sleep quality (34, 37).

More broadly, these findings should be interpreted within the context of substantial unmeasured or uncontrolled confounding factors across studies. Evidence from athletic populations indicates that sleep is highly sensitive to training load, session timing, competition demands, travel, and related lifestyle behaviors such as nutrition (50, 51). For example, higher training volumes have been associated with poorer sleep efficiency and increased fragmentation in swimmers, while periods of elevated physical and psychological stress in dancers have been linked to reduced sleep duration and greater nocturnal awakenings (52, 53). Similarly, increases in perceived training load in team sports such as Australian Rules Football have been shown to reduce total sleep time (54), while competition-related stress and scheduling constraints may further disrupt sleep through heightened physiological arousal and delayed recovery processes (55). Additionally, age-related differences, unfamiliar sleeping environments during travel, and variability in daily routines further complicate the interpretation of sleep outcomes (56, 57). Importantly, these factors are often intertwined with sport participation itself, making it difficult to isolate the specific effect of team sports on sleep quality. Taken together, the absence of systematic control or adjustment for training load, competition stress, exercise timing, and lifestyle variables represents an important limitation, and should be considered when interpreting the observed associations between team sport participation and sleep outcomes.

### 4.1 Limitations

This systematic review examined the effects of team sports on sleep quality; however, several limitations should be acknowledged. A primary constraint is the substantial clinical and methodological heterogeneity across the included studies, encompassing variations in study design, participant characteristics, and outcome measures. Differences in the type, intensity, and context of team sports (e.g., handball, volleyball, basketball, football, Zumba), as well as variability in populations (age, sex, health status, and fitness level), limit comparability and reduce the generalizability of the findings. In addition, most studies relied predominantly on self-reported sleep assessment tools, such as the PSQI, with limited use of objective measures (e.g., actigraphy). This reliance on subjective instruments introduces potential reporting and recall bias and may over- or underestimate true sleep changes, thereby warranting cautious interpretation of the observed associations. Furthermore, potential publication bias cannot be excluded, as studies reporting significant findings are more likely to be published and included. The methodological quality of the included studies also varied, with some lacking rigorous designs, standardized protocols, or appropriate control groups, which may further affect the reliability of the evidence base.

Moreover, although the decision not to conduct a meta-analysis was justified by the high degree of heterogeneity, this represents a notable limitation that substantially constrains the strength and precision of the conclusions. The absence of quantitative synthesis precludes estimation of pooled effect sizes and limits the ability to assess the magnitude, consistency, and robustness of the observed associations. Consequently, the findings of this review should be interpreted as qualitative and exploratory rather than definitive, and any inference regarding the effects of team sports on sleep quality should be considered tentative.

### 4.2 Strengths and recommendations for future research

This systematic review was conducted through comprehensive database searches, minimizing the likelihood of overlooking relevant studies. Although relatively few studies were included, they underwent rigorous quality assessment. Future research should prioritize long-term studies to assess the sustainability of sleep-related benefits from various team sports. Standardization in study designs—including clear control groups and uniform outcome measures—would enhance methodological consistency and improve comparability across studies. Ultimately, large-scale, longitudinal studies and meta-analyses involving diverse populations are essential to establish causal relationships between participation in team sports and sleep quality outcomes.

## 5 Conclusion

The findings of the present study indicate that participation in team sports—particularly soccer (football), basketball, handball, volleyball, and Zumba—is associated with improved sleep quality. Although, results regarding the effects of basketball, whether in the form of basketball games or wheelchair basketball, were inconsistent. However, considering the limited number of included studies and their methodological and population heterogeneity, these results should be regarded as preliminary and indicative rather than conclusive. While team-based physical activities may represent a promising and practical approach to supporting sleep health and overall well-being, the current evidence base remains constrained. Future research employing rigorous designs, larger sample sizes, and more homogeneous methodologies is warranted to clarify the direction, magnitude, and underlying mechanisms of this relationship across diverse populations.

## Data Availability

The tables in this published article contain the data generated or analyzed during this study. Requests to access these datasets should be directed to fa_ahmadif@yahoo.com.
